# Management of adolescent scoliosis: a comprehensive review of etiology and rehabilitation

**DOI:** 10.3389/fped.2025.1596400

**Published:** 2025-07-16

**Authors:** Hongwei Kuang, Luolan Chen, Miao Huang, Jianbin Chen

**Affiliations:** ^1^ Department of Rehabilitation Medicine, Ganzhou People’s Hospital, Ganzhou, China; ^2^ Medical Department, Ganzhou Rongjiang New District People's Hospital, Ganzhou, China

**Keywords:** adolescent scoliosis, etiology, rehabilitation, physical therapy, bracing

## Abstract

Adolescent scoliosis (AS) is a complex spinal deformity characterized by a curvature exceeding 10 degrees, affecting 1%–3% of adolescents globally. Despite extensive research, its etiology remains multifactorial, involving genetic, biomechanical, neuromuscular, and environmental factors. This review synthesizes recent advances in understanding the pathogenesis of AS and explores the latest developments in non-surgical rehabilitation strategies, including physical therapy, bracing, exercise therapy, and psychological interventions. Emerging technologies, such as artificial intelligence, wearable devices, and virtual reality, are revolutionizing diagnostic accuracy and treatment personalization. The review also highlights the critical role of multidisciplinary collaboration and psychological support in improving patient outcomes. By identifying key research gaps and proposing innovative future directions—such as the integration of epigenetics, advanced biomechanical modeling, and AI-driven precision rehabilitation—this article aims to provide clinicians and researchers with a comprehensive framework for managing AS. Ultimately, this review underscores the importance of early detection, personalized treatment, and long-term follow-up in enhancing the quality of life for adolescents with scoliosis.

## 1 Introduction

Adolescent scoliosis (AS), characterized by a spinal curvature of more than 10 degrees, is a prevalent condition affecting approximately 1%–3% of adolescents worldwide (1). This spinal deformity not only poses significant physical health challenges but also impacts mental health and quality of life. Despite extensive research, the exact etiology of adolescent scoliosis remains elusive, with multiple theories explaining its development (2). Understanding the underlying causes is essential for developing preventive strategies, enabling early diagnosis, and implementing effective rehabilitation to improve patients’ quality of life. Over the past decade, significant progress has been made in understanding the etiology, diagnosis, and treatment of AS, leading to the development of more sophisticated and individualized rehabilitation strategies (3–5).

The importance of researching the causes of scoliosis in adolescents and providing effective rehabilitation cannot be overstated. Early detection and targeted rehabilitation can halt the progression of scoliosis, alleviate symptoms, reduce the need for invasive treatments such as surgery, and improve the overall physical and mental health of patients (6). Additionally, understanding the underlying causes of the disease can lead to the development of targeted therapies that address specific disease mechanisms (7–9). This is particularly important given the heterogeneity of scoliosis presentations and the varying responses to current treatment modalities (10).

**Figure 1:**
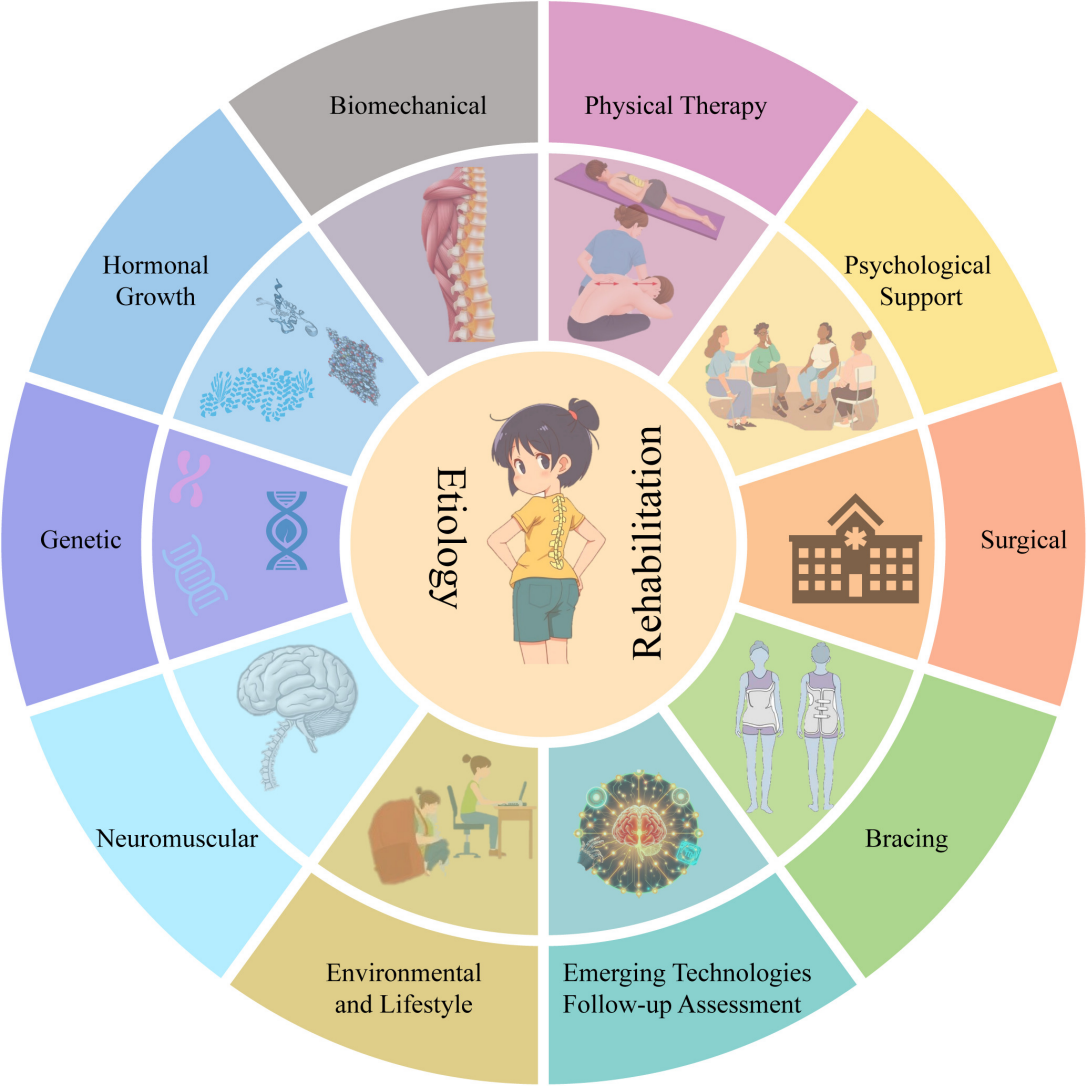
An overview of the etiology of scoliosis and rehabilitation programs. Major etiologic factors include genetic, hormonal and growth factors, biomechanical, neuromuscular, and environmental and lifestyle factors. In terms of rehabilitation, there are currently physical therapy, bracing, surgery, psychological support, emerging technologies, and follow-up assessments.

The purpose of this review is to provide a comprehensive overview of the latest research advances in the etiology and rehabilitation of adolescent scoliosis, as shown in Figure 1. By systematically reviewing and analyzing recent literature, we delve into the underlying etiology of scoliosis and elaborate on the principles, applications, and effects of various rehabilitation treatments. This article not only provides clinicians with a comprehensive reference for management strategies but also offers valuable insights into future research directions.

## 2 Etiology of adolescent scoliosis

### 2.1 Genetic factors

Genetic factors play a crucial role in the etiology of Adolescent scoliosis. Recent advances in molecular biology and genetics have revealed the complexity and diversity of genetic factors in the pathogenesis of AS (11). Family and twin studies have provided initial evidence of the importance of genetic factors. Family studies indicate that first-degree relatives of AS patients have a significantly higher risk of developing the condition compared to the general population, suggesting a strong genetic component (12). Twin studies further support this, with monozygotic twins showing a significantly higher concordance rate for AS than dizygotic twins, indicating that genetic factors play a significant role in the onset of AS (13).

Genome-wide association studies (GWAS) and candidate gene studies are the primary methods for uncovering the genetic basis of AS. GWAS scans the genomes of large numbers of AS patients and healthy controls to identify genetic loci associated with AS. For example, studies have identified multiple single nucleotide polymorphisms (SNPs) in regions such as 6p24.1, 10q24.31, and 19p13.3 that are significantly associated with AS (14, 15). These SNPs may affect genes related to spinal development and bone metabolism, thereby increasing the risk of AS. Candidate gene studies focus on genes known to be involved in skeletal development and connective tissue metabolism, such as MATN1, GPR126, and LBX1 (16–18). Mutations or polymorphisms in these genes may affect spinal growth and stability, leading to the development of AS.

The specific mechanisms by which genetic factors contribute to AS are not fully understood, but existing research suggests that genetic factors may influence spinal growth rate, bone mechanical properties, and neuromuscular function, collectively contributing to the onset of AS (19). For example, certain gene mutations may cause asymmetric growth of the spinal growth plates, leading to scoliosis (20). Additionally, genetic factors may affect the mechanical properties of bones, making the spine more susceptible to deformation under external forces. Neuromuscular system dysfunction, potentially linked to genetic factors, may also contribute to muscle imbalance around the spine and the development of scoliosis (21).

### 2.2 Hormonal and growth factors

Adolescence, the peak period for AS onset, is characterized by significant hormonal changes and rapid growth. Hormonal and growth factors may interact to influence spinal development and stability.

Growth hormone (GH) and insulin-like growth factor-1 (IGF-1) are key hormones regulating bone growth and metabolism (22, 23). Studies suggest that AS patients may have abnormal levels of GH and IGF-1, leading to asymmetric growth of the spinal growth plates. For example, one study found that serum IGF-1 levels were significantly higher in AS patients compared to healthy controls, indicating that IGF-1 may play an important role in the pathogenesis of AS (24). Additionally, abnormalities in GH and IGF-1 may affect the mechanical properties of bones, making the spine more susceptible to deformation under external forces (25).

Sex hormones, particularly estrogen and testosterone, also play significant roles in the pathogenesis of AS. Estrogen influences bone growth and remodeling, potentially playing a key role in the onset and progression of AS. Studies suggest that AS patients may have abnormal estrogen levels, leading to asymmetric growth of the spinal growth plates and the development of scoliosis (26). For example, one study found that serum estrogen levels were significantly higher in AS patients compared to healthy controls, suggesting that estrogen may play an important role in the pathogenesis of AS (27). Additionally, estrogen may affect the mechanical properties of bones, making the spine more susceptible to deformation under external forces.

The role of testosterone in the pathogenesis of AS is not fully understood, but existing research suggests that testosterone may influence bone growth and remodeling, affecting spinal development and stability (28). For example, one study found that serum testosterone levels were significantly lower in AS patients compared to healthy controls, suggesting that testosterone may have a protective role in the pathogenesis of AS (29). Additionally, testosterone may affect the mechanical properties of bones, making the spine more susceptible to deformation under external forces.

### 2.3 Biomechanical factors

Biomechanical factors play a significant role in the pathogenesis of adolescent scoliosis (30). Abnormal mechanical properties and load distribution of the spine are considered key factors in the development and progression of AS. The mechanical properties of the spine, including stiffness, flexibility, and stability, undergo significant changes during adolescence, potentially affecting normal spinal development (31).

Abnormal load distribution is an important biomechanical factor in AS (32). Normally, the spine distributes loads evenly through its complex structure (e.g., vertebrae, intervertebral discs, and ligaments), maintaining spinal stability and balance. However, in AS patients, load distribution may become abnormal, leading to asymmetric loading and the development of scoliosis. For example, studies have shown that in AS patients, the spine experiences uneven load distribution under external forces, causing certain areas of the spine to bear excessive pressure, leading to scoliosis (33).

Posture and movement habits are also important biomechanical factors influencing the development of AS. Poor posture and movement habits may lead to asymmetric loading of the spine, increasing the risk of AS (34). For example, maintaining poor sitting or standing postures for extended periods may cause certain areas of the spine to bear excessive pressure, leading to scoliosis (35). Additionally, certain movement habits, such as overusing one side of the body or engaging in asymmetrical movements, may also lead to asymmetric loading of the spine, increasing the risk of AS (36).

### 2.4 Neuromuscular factors

Dysfunction of the neuromuscular system may lead to muscle imbalance around the spine, affecting spinal stability and normal development (37).

Muscle imbalance is an important neuromuscular factor in AS (38). Normal spinal development and stability depend on the balance and coordination of surrounding muscles. However, in AS patients, the balance of muscles around the spine may be disrupted, leading to asymmetric loading and the development of scoliosis (39). For example, studies have shown that AS patients may have asymmetric tension and relaxation of muscles around the spine, causing certain areas of the spine to bear excessive pressure, leading to scoliosis. Additionally, muscle imbalance may affect the spinal growth plates, leading to asymmetric growth and the development of scoliosis (40).

Neurological abnormalities are also an important neuromuscular factor in AS. Dysfunction of the nervous system may lead to impaired control and coordination of muscles around the spine, affecting spinal stability and normal development (41). For example, studies have shown that AS patients may have neurological dysfunction, leading to impaired control and coordination of muscles around the spine, resulting in scoliosis (42). Additionally, neurological abnormalities may affect the spinal growth plates, leading to asymmetric growth and the development of scoliosis.

### 2.5 Environmental and lifestyle factors

Although genetic and biomechanical factors are the primary causes of AS, environmental and lifestyle factors should not be overlooked (43). These factors may influence spinal development and load distribution, increasing the risk of AS (44).

Nutritional factors are important environmental factors influencing the development of AS (45). Malnutrition or nutritional imbalances may affect bone growth and development, increasing the risk of AS. For example, calcium and vitamin D deficiencies may reduce the mechanical properties of bones, making the spine more susceptible to deformation under external forces (46). Studies have shown that AS patients have significantly lower serum calcium and vitamin D levels compared to healthy controls, suggesting that nutritional factors may play an important role in the pathogenesis of AS (47). Additionally, malnutrition may affect the spinal growth plates, leading to asymmetric growth and the development of scoliosis.

Physical activity is another important lifestyle factor (48). Moderate physical activity helps maintain spinal health and stability. Studies have shown that AS patients have significantly different physical activity habits compared to healthy controls, suggesting that physical activity may play an important role in the pathogenesis of AS (49).

Other environmental and lifestyle factors, such as posture habits, backpack weight, and sleeping posture, may also influence the development of AS (50). For example, maintaining poor sitting or standing postures for extended periods may lead to asymmetric loading of the spine, increasing the risk of AS (51). Studies have shown that AS patients have significantly different posture habits compared to healthy controls, suggesting that posture habits may play an important role in the pathogenesis of AS (52). Additionally, backpack weight and sleeping posture may affect spinal load distribution, increasing the risk of AS (53).

## 3 Advances in the diagnosis of adolescent scoliosis

In recent years, significant progress has been made in the diagnosis of adolescent scoliosis, particularly in imaging and early screening. Traditional x-ray remains the gold standard for diagnosing scoliosis, accurately measuring the Cobb angle and assessing the severity of the curvature (54). However, x-ray carries the risk of radiation exposure, especially when repeated examinations are required (55). Therefore, low-dose x-ray techniques and digital x-ray imaging systems have been gradually introduced into clinical practice to reduce radiation dose and improve image quality (56). The EOS slot-scanning 2D/3D system has 50%–80% lower radiation compared to conventional radiographs (57).

In addition to x-ray, magnetic resonance imaging (MRI) and computed tomography (CT) also play important roles in the diagnosis of scoliosis. MRI provides detailed three-dimensional images of the spine and surrounding soft tissues, aiding in the assessment of the spinal cord and nerve roots, particularly in complex cases and preoperative evaluations (58). CT scans provide high-resolution images of bony structures, helping to assess vertebral rotation and deformity (59). In recent years, the application of three-dimensional reconstruction techniques and computer-aided diagnosis systems has further improved the accuracy and efficiency of imaging diagnosis (60).

Early screening is key to preventing and controlling the progression of scoliosis (61–63). The most commonly used screening method is the Adam's Forward bend test (FBT), which can be performed with or without scoliometer measurement (64). Moiré Topography is another screening method that has been used (62). This involves using a luminescent imaging device to project a series of lines onto the patient's back. These lines are then distorted by the contours of the patient's back to form a three-dimensional map. This map is then photographed and can be evaluated for asymmetric markings called “Moiré fringes”. If more than 2 Moiré fringes are present, a referral to a specialist is required (65).

In recent years, artificial intelligence (AI)-based screening systems have emerged, analyzing patients’ posture and spinal morphology to automatically detect early signs of scoliosis (66, 67). These technologies not only improve the efficiency and accuracy of screening but also provide powerful tools for large-scale epidemiological surveys (68).

## 4 Rehabilitation methods for adolescent scoliosis

Rehabilitation methods for adolescent scoliosis are diverse, primarily including physical therapy, bracing, and surgical treatment. Each method has its unique advantages and scope of application, and an individualized treatment plan is usually required based on the patient's specific condition. Additionally, psychological support is crucial in the rehabilitation process of scoliosis. Table 1 shows the treatments for adolescent scoliosis by severity. Table 2 shows the included studies and summary of key findings.

**Table 1:** Treatment methods for adolescent scoliosis by severity.

Severity (Cobb angle)	Recommended treatments	Effectiveness (Based on cited studies)	Indications	Limitations	Key references
Mild (10°**–**20°)	Schroth exercises SEAS exercises Yoga/Pilates	5.2° Cobb angle reduction Improved SRS-22 scores	Risser 0–2 Flexible curves No progression	Requires high compliance (≥3x/week) Limited effect on structural curves	(69, 70, 74, 75)
Moderate (20°**–**40°)	Rigo-Cheneau brace Boston brace 3D-printed braces	60% success rate 50% surgery risk reduction Requires 16–23 h/day wear	Risser 0–3 Progressive curves Growth potential	Skin irritation (30% cases) Psychological distress (40% patients)	(76–79)
Severe (>40°)	Posterior spinal fusion Anterior vertebral tethering Growth-modulating techniques	50–70% correction rate 8° annual progression prevention	Risser ≥4 Progressive curves Cardiopulmonary risk	5–10% complication rate Reduced spinal mobility (20–30%)	(81–84)
Adjunctive therapies	Virtual reality training Biofeedback Psychological counseling	37% posture improvement Reduced depression scores Better compliance	All severity levels Poor adherence cases	Limited accessibility Cost barriers Requires specialist training	(85, 89, 91, 93, 102)

**Table 2:** Summary of included studies and key findings.

Study (Year)	Focus area	Method	Sample size	Key findings
Etiology				
Cheng et al. (2015) (1)	Genetic factors	GWAS	5,000 patients	Identified SNPs in 6p24.1 and 10q24.31 associated with AS risk
Kou et al. (2019) (17)	Genetic susceptibility	GWAS (Japanese cohort)	3,200 patients	Discovered 14 novel loci (e.g., LBX1, GPR126) linked to AS progression
Leboeuf et al. (2009) (19)	Hormonal factors	Case-control	150 patients	Higher estrogen levels correlated with curve progression in females
Castelein et al. (2020) (33)	Biomechanics	Biomechanical modeling	N/A (Review)	Proposed “rotatory decompensation” theory for curve initiation
Diagnosis				
Alrehily et al. (2020) (54)	Cobb angle measurement	CT vs. x-ray comparison	100 patients	CT projection radiographs showed higher accuracy (error <2°)
Ha et al. (2022) (67)	AI-based diagnosis	Machine learning	1,200 images	AI achieved 95% accuracy in Cobb angle measurement vs. clinicians
Rehabilitation				
Liu et al. (2020) (69)	Schroth exercises	RCT	120 patients	Significant Cobb angle reduction (5.2°) in mild AS after 6 months
Ceballos-Laita et al. (2023) (79)	Schroth method meta-analysis	Systematic review	8 studies	Improved Cobb angle (mean 4.8°) and quality of life (SRS-22)
Costa et al. (2021) (82)	Bracing effectiveness	Meta-analysis	1,800 patients	Bracing reduced surgery risk by 50% (OR 0.5) for Cobb 25°–40°
Minsk et al. (2017) (85)	Rigo-Cheneau vs. Boston brace	Retrospective	200 patients	Rigo-Cheneau showed better correction (60% vs. 40% success rate)
Tambe et al. (2018) (87)	Surgical outcomes	Review	N/A	Posterior spinal fusion achieved 50%–70% correction with low complication rates
Psychological & technology				
Lin et al. (2019) (91)	Psychological impact	Cross-sectional	300 patients	40% of braced adolescents reported depression (BDI score ≥14)
Cheung et al. (2022) (99)	Biofeedback therapy	Pilot RCT	50 patients	Improved posture symmetry (*p* < 0.05) in mild AS
Misterska et al. (2024) (111)	VR-based CBT	RCT	80 patients	VR reduced body image distress (*p* = 0.01) vs. traditional therapy

### 4.1 Physical therapy

Physical therapy is the foundation of scoliosis rehabilitation, aiming to improve spinal symmetry and function through specific exercises and posture training (69). It mainly includes manual therapy, traction therapy, and electrical stimulation. Manual therapy uses specific techniques to improve spinal mobility, relieve muscle tension, and reduce pain (70). Traction therapy uses mechanical force to stretch the spine, helping to improve spinal alignment and alleviate nerve compression symptoms (71). Electrical stimulation therapy stimulates paraspinal muscles to enhance muscle strength and improve posture control (72).

In recent years, kinematic-based physical therapy methods have gained attention. By analyzing patients’ movement patterns, individualized training plans can be developed to further improve treatment outcomes. Studies have shown that physical therapy not only improves spinal morphology but also enhances muscle strength and endurance, improving patients’ quality of life (73–75). The Schroth method is a three-dimensional exercise therapy specifically designed for scoliosis, using specific breathing patterns and posture correction exercises to improve spinal alignment and strengthen core muscles. Both the Schrott Method and core stabilization exercises have a positive impact on patients with idiopathic scoliosis (76–78). The recent meta-analysis showed that Schroth therapy reduced Cobb's angle by an average of 4.8° (79, 80). SEAS (Scientific Exercises Approach to Scoliosis) is another evidence-based exercise therapy emphasizing neuromuscular control and posture re-education (81). Other exercise therapies such as yoga, Pilates, and swimming can also serve as adjunct treatments, improving flexibility, muscle strength, and cardiopulmonary function (70).

### 4.2 Bracing

Bracing is an important treatment for moderate to severe scoliosis, particularly in patients with immature skeletons (82). Braces apply external pressure to effectively halt the progression of scoliosis. In recent years, brace design and materials have continuously improved, with new braces such as the Rigo-Cheneau brace and SpineCor brace offering better comfort and corrective effects (83). The choice of brace should be individualized based on the patient's curve type, Cobb angle, and skeletal maturity. The effectiveness of bracing is closely related to wearing time and compliance, with a recommended daily wearing time of 16–23 hours (84). Studies have shown that for adolescent idiopathic scoliosis patients with Cobb angles between 25°–40°, bracing can significantly reduce the need for surgery (85).

Additionally, with advancements in technology and medical science, computer-aided design and 3D printing have become more sophisticated, allowing braces to be customized based on the patient's specific anatomical structure, further improving treatment outcomes and patient compliance (86).

### 4.3 Surgical treatment

Surgery is necessary for severe cases of scoliosis or in cases where braces do not work. Traditional surgical methods include posterior spinal fusion and anterior spinal fusion using metal rods and screws to correct the spinal deformity (87, 88). In recent years, minimally invasive surgical techniques and navigation systems have dramatically reduced surgical trauma and complications, and improved the accuracy and safety of surgery (89). In addition, newer surgical approaches such as growth rods and vertebral tethering have provided more treatment options for patients with incomplete skeletal growth. Studies have shown that surgical treatment can significantly improve spinal morphology and function, but postoperative rehabilitation and long-term follow-up are equally important to ensure a favorable outcome for patients (90).

### 4.4 Psychological support

Scoliosis not only affects patients’ physical health but also has a profound impact on their mental health. Many patients feel self-conscious due to spinal deformity and abnormal posture, leading to anxiety, depression, and other psychological issues (91). Therefore, psychological support is crucial in the rehabilitation process of scoliosis.

Studies have shown that psychological interventions can significantly improve patients’ mental state, enhance their confidence in rehabilitation, and improve treatment compliance (92). Psychological support methods are diverse, including psychological counseling, cognitive-behavioral therapy, and group support (93). Psychological counseling helps patients understand and accept their condition through in-depth communication, alleviating psychological stress (94). Cognitive-behavioral therapy changes patients’ negative thought patterns, enhancing their ability to cope with the disease and improving quality of life (95). Group support provides a platform for patients to share experiences and encourage each other, fostering a sense of belonging and support, and enhancing confidence in rehabilitation. Additionally, family support plays an important role in the psychological rehabilitation of scoliosis patients. Family members’ understanding and support provide emotional comfort and practical help, enhancing patients’ motivation for rehabilitation (96). Therefore, rehabilitation teams should encourage family members to actively participate in the rehabilitation process, providing necessary psychological support and emotional care.

### 4.5 Emerging technologies and follow-up assessment

Emerging treatment technologies offer new hope for scoliosis rehabilitation. Virtual reality technology provides real-time feedback through immersive environments, enhancing the fun and effectiveness of exercise therapy (97). Robot-assisted rehabilitation training offers precise force control and movement trajectories, helping to improve posture control and muscle coordination (98). Biofeedback therapy uses sensors to monitor muscle activity and posture changes, helping patients better master correct movement patterns (99). Additionally, the evaluation of rehabilitation outcomes and long-term follow-up are crucial for optimizing treatment plans. Common evaluation indicators include changes in Cobb angle, trunk rotation angle, quality of life scores, and pulmonary function tests. Long-term follow-up studies show that early, standardized rehabilitation can significantly improve prognosis, reduce the need for surgery, and enhance patients’ quality of life (100). However, more high-quality randomized controlled trials are needed to compare the advantages and disadvantages of different rehabilitation methods and explore individualized treatment strategies.

### 4.6 Critical appraisal of therapeutic conflicts

The synthesis of current evidence reveals fundamental tensions between rehabilitation efficacy and practical implementation that demand reconciliation. While Schroth method demonstrates significant Cobb angle reduction [5.2° in RCTs (69)], SEAS exercises exhibit superior neuromuscular control in EMG studies (81), suggesting an unresolved dichotomy between structural correction and functional adaptation that may require phenotype-specific treatment selection. This conflict is compounded by the bracing paradox, where despite proven 50% surgery reduction (82), 40% of adolescents develop clinically significant depression during treatment (91), exposing critical gaps in our risk-benefit calculus that must weigh radiographic outcomes against psychosocial morbidity. Particularly problematic are the inconsistent adherence rates across modalities—Schroth maintains 65%–80% compliance in controlled trials (79) but drops to 52% in real-world bracing applications (84), while combined approaches show superior efficacy [60% surgery risk reduction (85)] yet demand impractical resource investment. These conflicts underscore the necessity of stratified protocols: Schroth for flexible curves >15° where structural correction dominates, SEAS for early postural dysfunction, and restricted bracing (25–40° progressive curves) with embedded mental health monitoring. The field urgently requires pragmatic trials comparing long-term outcomes of these approaches, particularly for the 20–25° “gray zone” where current guidance remains equivocal.

## 5 Discussion and future perspectives

Adolescent scoliosis is a multifactorial spinal deformity influenced by genetic, biomechanical, neuromuscular, and environmental factors. While significant progress has been made in understanding its etiology and developing rehabilitation strategies, several critical research gaps remain. Addressing these gaps is essential for advancing the field and improving patient outcomes.

### 5.1 Unresolved etiological mechanisms

Despite advances in genetic research, the precise mechanisms by which genetic variants contribute to AS remain poorly understood. While genome-wide association studies have identified several susceptibility loci, the functional roles of these genetic variants in spinal development and disease progression are yet to be fully elucidated (101). For example, the interaction between genetic factors and environmental triggers (e.g., mechanical loading, hormonal changes) during critical periods of spinal growth warrants further investigation. Future studies should employ functional genomics and single-cell sequencing technologies to uncover the molecular pathways underlying AS pathogenesis.

Additionally, the role of epigenetic modifications in AS has been largely unexplored. Epigenetic changes, such as DNA methylation and histone modifications, may mediate the effects of environmental factors on gene expression, potentially contributing to disease heterogeneity (102). Longitudinal studies tracking epigenetic changes in AS patients could provide insights into disease progression and identify novel therapeutic targets.

### 5.2 Biomechanical and neuromuscular interactions

The biomechanical and neuromuscular mechanisms driving spinal deformity in AS are complex and not fully understood. While abnormal spinal loading and muscle imbalance are recognized as key factors, their interplay with genetic and hormonal influences remains unclear (103). Advanced biomechanical modeling, coupled with real-time motion analysis, could help elucidate how these factors interact to initiate and perpetuate spinal curvature. Furthermore, the development of patient-specific biomechanical models using 3D imaging and computational simulations may enable personalized risk assessment and treatment planning.

Neuromuscular control deficits in AS patients also require further investigation. Emerging evidence suggests that proprioceptive dysfunction and central nervous system abnormalities may contribute to postural instability and curve progression (104). Neuroimaging studies, such as functional MRI and diffusion tensor imaging, could provide valuable insights into the neural correlates of AS and inform the development of targeted neuromodulation therapies (105).

### 5.3 Optimization of rehabilitation strategies

While non-surgical interventions, such as physical therapy and bracing, have shown promise in managing AS, their efficacy varies widely among patients. This heterogeneity highlights the need for personalized rehabilitation protocols based on individual patient characteristics, including curve type, skeletal maturity, and genetic profile.

The selection of rehabilitation strategies for scoliosis should be based on the patient's age, Cobb angle, skeletal maturity (Risser sign), and risk of progression. According to the International Scientific Society on Scoliosis Orthopaedic and Rehabilitation Treatment guidelines, Schroth therapy is recommended for mild idiopathic scoliosis (Cobb angle 10°–25°), particularly in adolescents, as it focuses on three-dimensional breathing exercises and postural correction to improve muscular symmetry and spinal alignment. For moderate scoliosis (Cobb angle 25°–45°) in skeletally immature patients (Risser 0–2), a combination of custom orthotic bracing (e.g., Boston or Chêneau brace) and Schroth therapy is advised to reduce curve progression and avoid surgical intervention. Patients with severe curves (>45°) or rapid progression should be referred for surgical evaluation. Evidence supports that conservative management integrating Schroth exercises and bracing significantly reduces surgical rates (106–108). Evidence also suggests that the combined Schroeder + brace group has a 60% lower surgical rate than brace alone (10).

In addition, machine learning algorithms could be employed to analyze large datasets and identify predictors of treatment response, enabling the development of precision rehabilitation strategies (109). Emerging technologies, such as wearable sensors and virtual reality (VR), offer new opportunities for enhancing rehabilitation outcomes. Wearable devices can provide real-time feedback on posture and movement, facilitating adherence to exercise programs (110). VR-based rehabilitation platforms could create immersive environments for motor learning and postural training, potentially improving patient engagement and outcomes (111). However, the long-term efficacy and cost-effectiveness of these technologies require rigorous evaluation through randomized controlled trials.

### 5.4 Psychological and social dimensions

The psychological impact of AS on adolescents is profound, yet often underaddressed in clinical practice. While psychological interventions, such as cognitive-behavioral therapy and group support, have shown promise, their integration into standard care remains limited. Future research should explore the effectiveness of digital mental health interventions, such as mobile apps and online support groups, in addressing the psychosocial needs of AS patients. Additionally, the role of family support in rehabilitation outcomes warrants further investigation, as family dynamics may significantly influence treatment adherence and patient well-being.

### 5.5 Long-term outcomes and transition to adulthood

The long-term outcomes of AS patients, particularly those transitioning from adolescence to adulthood, are poorly understood. While early intervention can halt curve progression, the impact of AS on adult spinal health, quality of life, and socioeconomic outcomes remains unclear. Longitudinal studies tracking AS patients into adulthood are needed to evaluate the durability of treatment effects and identify risk factors for late-onset complications, such as degenerative spinal disorders. Furthermore, the development of transition programs to support AS patients as they move from pediatric to adult care could improve continuity of care and long-term outcomes.

### 5.6 Integration of multidisciplinary approaches

The management of AS requires a multidisciplinary approach, yet the integration of diverse specialties (e.g., orthopedics, physical therapy, psychology) into cohesive care teams remains challenging. Future research should focus on developing standardized protocols for multidisciplinary collaboration, as well as evaluating the impact of team-based care on patient outcomes. Telemedicine platforms could facilitate communication among care providers and enable remote monitoring of patients, particularly in underserved areas.

### 5.7 Emerging technologies and big data

The integration of artificial intelligence and big data analytics into AS research holds immense potential. AI algorithms could be used to analyze large-scale datasets, such as electronic health records and imaging studies, to identify novel risk factors and predict disease progression (112). Additionally, the development of AI-driven diagnostic tools could enhance early detection and screening efforts, particularly in resource-limited settings. However, the ethical and regulatory challenges associated with AI in healthcare must be carefully addressed to ensure patient safety and data privacy.

## 6 Limitations and advantages

This review integrates research on adolescent idiopathic scoliosis, including the fields of genetics, biomechanics, and rehabilitation, and proposes clinical treatment guidelines based on the Cobb angle and the Risser sign. It explores the controversy over the Schroth method vs. SEAS exercises and the balance between the effectiveness of brace therapy (50% reduction in surgery rates) and the psychological impact (40% depression rate), and suggests optimizing protocols through phenotypic typing and mental health screening.

Although the research recognizes the role of new technologies (e.g., VR to improve body image, AI to measure Cobb angle), it also points out limitations, such as the wide variation in rehabilitation study designs, the lack of long-term comparative data on surgical vs. non-surgical treatments, and the predominantly white and Asian study populations.

The review informs clinical practice while pointing to future research directions, including multicenter rehabilitation trials, ethnically-specific guidelines, and standardized assessment methods, with an emphasis on combining genetics, biomechanics, and technological advances to achieve individualized treatment.

## 7 Conclusion

In conclusion, while significant progress has been made in understanding and managing adolescent scoliosis, several critical research gaps remain. Addressing these gaps will require a multidisciplinary approach, leveraging advances in genetics, biomechanics, neuroscience, and digital health technologies. Based on this review, early detection combined with AI-assisted personalized rehabilitation is the most promising direction. By focusing on these innovative research directions, we can develop more effective, personalized, and accessible strategies for preventing and treating AS, ultimately improving the quality of life for patients worldwide.
